# A mixed-methods study protocol: Perinatal depression screening systems and outcomes in obstetrics clinics

**DOI:** 10.1371/journal.pone.0319181

**Published:** 2025-03-21

**Authors:** Rachel Vanderkruik, Marlene P. Freeman, Margaret Gaw, Audrey R. L. Reuman, Maya Verghese, Courtney C. Louis, Michael Jellinek, Stephen Bartels, Lee S. Cohen

**Affiliations:** 1 Massachusetts General Hospital, Ammon-Pinizzotto Center for Women’s Mental Health, Boston, Massachusetts, United States of America; 2 Harvard Medical School, Boston, Massachusetts, United States of America; 3 The Mongan Institute, Massachusetts General Hospital, Harvard Medical School, Boston, Massachusetts, United States of America; University of Zurich, SWITZERLAND

## Abstract

Perinatal depression (PND) is an underrecognized and underdiagnosed public health issue with long-term adverse impacts on birthing parents and their children. While obstetrics practices are increasingly encouraged to use existing evidence-based screening tools, there is little data describing the extent to which screening practices and subsequent referrals to care are implemented in clinical settings. The Screening and Treatment Enhancement for Perinatal Depression (STEPS for PPD) study aims to characterize PND screening and referral procedures and identify areas for system improvements. We describe a protocol for an observational study, guided by implementation science frameworks, examining the role of embedded perinatal social workers in managing PND across Mass General Brigham system obstetrics clinics. Our mixed-methods approach integrates qualitative and quantitative data from a variety of sources, including electronic health records, patient-reported surveys, and qualitative interviews, to capture complex screening and referral practices across a large academic medical system. We aim to characterize nuances within the screening and referral system and identify barriers and facilitators to care to inform future hybrid-implementation effectiveness research and improve patient outcomes.

## Introduction

Perinatal depression (PND) is defined as a major depressive episode with onset during pregnancy or the first four weeks following childbirth [1,2]. Though PND is one of the most common complications associated with the perinatal period, with a mean prevalence of 11.5% [3], it remains under-recognized; in nationally representative data of pregnant individuals with depressive symptoms, PND remained undiagnosed in two thirds (65.9%) and untreated in half (49.6%) of cases [3–5]. Untreated PND may have long-lasting negative consequences on families, including compromised parent-infant bonding [4,6,7], lower rates of breastfeeding [8], and developmental disorders among children of those with the condition [9]. The national estimated annual cost across two generations per patient with untreated PND is $22,647 [10].

The “Perinatal Depression Treatment Cascade” describes common challenges along the path of clinical recognition to treatment for perinatal depression. The Cascade highlights the necessity of several steps to ensure adequacy of treatment, including a patient a) entering the health care system, b) having depressive symptoms detected via an evidence-based screening method, c) having treatment initiated, d) receiving an effective treatment trial, and e) receiving follow-up assessment to ensure adequate remission of symptoms [11]. To our knowledge, very few individuals receive all these domains of care, and research describing outcomes across each of these cascade components has been sparse.

As a result, many countries, including the United States, have called for efforts to recognize the contribution of mental illness to pregnancy-related death and disease and to bolster research, programs, and policies to support perinatal mental health [12]. In 2016, for example, the US Preventative Services Task Force recommended that health care systems implement universal PND screening to enable accurate diagnosis and adequate treatment and follow-up care [13]. Relatedly, the American Academy of Pediatrics recommends parental PND screening at infant well-child visits up to 6 months postpartum [14], and in 2023, the American College of Obstetrics and Gynecology (ACOG) released recommendations to screen for depression using standardized, validated instruments at least twice during pregnancy (at the initial prenatal visit and later) and in the postpartum [15]. In light of these recommendations, many states in the U.S. have embraced universal screening programs for PND, often using the Edinburgh Postnatal Depression Scale (EPDS), a ten-item, patient-completed depressive symptoms rating scale validated for the detection and assessment of perinatal depression and most often implemented in obstetrics (OB) settings [16,17]. There is substantial literature supporting the utility of screening tools (e.g., the EPDS) for detection and diagnosis of perinatal depression [18–20]. Importantly, the use of PND screening tools should be accompanied by symptom management and follow-up by the treating healthcare team [21,22]; 2023 ACOG guidelines specifically recommend “mental health screening be implemented with systems in place to ensure timely access to assessment and diagnosis, effective treatment, and appropriate monitoring and follow-up based on severity” [15].

Despite these recommendations, perinatal depression screening rates vary; in one study of 35 clinics, rates ranged from as low as 24% to as high as 100% [23]. Rates may also be influenced by existing social inequities: publicly insured patients, non-native English speakers [23], and individuals living in poverty [24] appear less likely to receive evidence-based PND screening than the privately insured, native English speakers, and higher income individuals, respectively. African American (adjusted odds ratio, aOR =  0.81), Asian (aOR =  0.64), or otherwise non-white (Native American, multi-racial, aOR =  0.44) patients are also less likely to be screened for postpartum depression than their white counterparts [23].

Documented barriers to screening may exacerbate these inequities across multiple ecological levels [25,26]. Hu and colleagues conducted a qualitative systematic review of barriers to and facilitators of psychological help-seeking behaviors among perinatal people with depressive symptoms and mapped their findings onto the Consolidated Framework for Implementation Research (CFIR). They found that, on the individual level, perceptions of cultural stigma or parenthood norms may influence patient willingness to disclose symptoms. Healthcare practitioners, meanwhile, may not recognize the severity of symptoms; may experience language barriers; or may encounter a shortage, either due to limited availability or insurance restrictions, of mental healthcare providers to whom to refer patients. Healthcare costs and other financial barriers may further impede access to care [25].

Therefore, although the need for PND screening and referral has been established and clinics are increasingly adopting policies and procedures to address it, a variety of multi-level barriers may limit the impact of such initiatives. Further, both research on long-term patient outcomes following screening as well as guidance on effective implementation procedures are lacking [25,27,28]. This lack of clarity has produced a wide range of implemented screening (e.g., frequency, tracking, score cutoffs) and referral (e.g., service integration, time to follow-up) practices within OB clinics. Such variability may impact effectiveness and contribute to uncertainty around the merits of screening and referral programs. In fact, some have questioned the utility of universal screening for perinatal depression because of the limited available data pertaining to its effectiveness and outcomes [29]. We, on the other hand, argue that universal screening is a critical component of detection for PND, and that more research intended to identify effective implementation strategies is needed to ensure perinatal patients receive adequate care after screening and ultimately return to wellness [16]. We developed the Screening and Treatment Enhancement for Perinatal Depression (STEPS for PPD) project to address these gaps in the universal PND screening literature [30].

Observational studies can provide insights into real-world processes as well as estimates of treatment rates and clinical outcomes within healthcare delivery systems [31]. The STEPS for PPD project aims to observe the complexities of real-world, multicomponent PND screening and referral processes across several OB clinics within the Massachusetts General Brigham (MGB) healthcare system that have implemented routine prenatal and postpartum depression screening using the EPDS. By establishing a detailed understanding of PND management practices within OB care, our project is well-positioned to evaluate patient outcomes and inform the improvement of services [32].

The goals of the STEPS for PPD study are thus to descriptively characterize differences in existing protocols, evaluate clinical outcomes, and assess factors that impact screening and referral across multiple sites. Importantly, our goal is not to implement and assess fidelity to a single protocol, as many clinics have adopted their own. Rather, we explore factors that influence screening and referral processes within existing protocols. Our mixed-methods evaluation of these systems is guided by two implementation science frameworks: Reach, Effectiveness, Adoption, Implementation, and Maintenance (RE-AIM) [33] and the Consolidated Framework for Implementation Research (CFIR) [34]. These frameworks help us to identify *who* is receiving screening, referral, and treatment (i.e., intervention “reach”), treatment received and *change in depressive symptoms* among patients (i.e., intervention “effectiveness”), as well as *how* screening and referral procedures are implemented across clinics (i.e., intervention “implementation”).

Assessment of screening procedures and outcomes at each of the clinics can help to highlight barriers and facilitators to implementation of screening and referral programs and inform strategies to address identified barriers [35]. The use of both qualitative and quantitative data is common in health services research given the need to investigate complex, multilevel processes and systems [36,37]. Whereas quantitative methods often address research questions about magnitude of effects or causality, qualitative methods can help to understand patient experiences and why or how a finding or phenomenon occurs. Mixed methods research thus leverages the strengths of both quantitative and qualitative approaches and has been described as providing “an innovative approach for addressing contemporary issues in health services” [38]. We integrate quantitative and qualitative data through an explanatory sequential design [37,38] and in the reporting of our findings, described further in the analytic approach below.

## Methods

### Study design overview

This observational study, approved by the MGB Institutional Review Board (Protocol # 2020P003857), evaluates the use and outcomes of screening with the EPDS for perinatal depression across four obstetric care sites within the MGB healthcare system. Participating clinics have adopted their own protocols whereby patients with elevated EPDS scores are referred to a perinatal social worker (SW) for further assessment, treatment, and/or care navigation. Each clinic implements a multicomponent intervention that includes 1) screening with the EPDS, 2) referral to an internal perinatal SW, and 3) outreach by the SW. Electronic health record (EHR) data stored in EPIC Systems software are used to identify pregnant and postpartum individuals with elevated EPDS scores receiving obstetric care from participating clinics.

**Fig 1:**
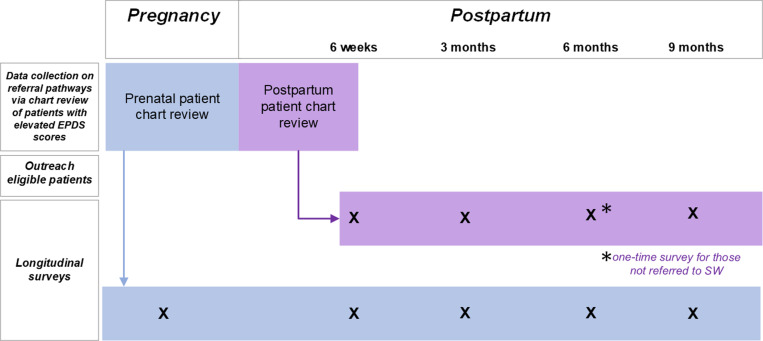
Study participant flow across pregnancy and the postpartum.

The patient cohort can be stratified into sub-groups based on a) when (i.e., during pregnancy or the postpartum) the individual presented with elevated depressive symptoms as per EPDS score and b) whether or not they were subsequently referred to an embedded perinatal SW. Research staff invite any patient with an elevated score at a routine prenatal or postpartum visit who was subsequently referred to SW to submit self-report data through completion of a series of surveys spanning the perinatal period. Pregnant participants complete five surveys: one during pregnancy and one each at 6 weeks, 3 months, 6 months, and 9 months postpartum. Postpartum participants complete the latter four (postpartum) surveys. Outreach to any eligible pregnant and postpartum patient begins within one week of the elevated score being documented in the EHR. The eligibility of patients referred following non-routine screening (e.g., EPDS administration outside of the 6-week postpartum visit) is discussed on a case-by-case basis by the project team. Due to observed heterogeneity of screening and referral practices in real-world contexts and in order to better understand why postpartum individuals with elevated EPDS scores may not be referred to internal social work, our team also added a one-time assessment for postpartum individuals who are *not* referred to social work after an elevated EPDS score or who do not have an EPDS score on record for the postpartum visit. This group is invited to complete a one-time survey approximately six months after their routine 6-week postpartum visit. See Fig 1 for a visual of assessment timepoints.

The study enrolls participants from four hospital-based sites across the MGB system: Newton Wellesley Hospital (NWH; approximately 4,500 births per year), North Shore Medical Center (NSMC; approximately 850 births per year), Massachusetts General Hospital (MGH, approximately 3,500 births per year), and Brigham and Women’s Hospital (BWH, approximately 6,200 births per year). Each site serves patients from several clinics (i.e., NWH is comprised of three clinics and MGH of six) covering a range of geographic areas and patient populations in the Boston Metro area. English-speaking adults (≥18 years old) who are receiving obstetric care from one of the participating sites and have an elevated EPDS screen (e.g., total score at NWH, BWH, MGH: ≥ 10, NSMC: ≥ 9 OR who endorse thoughts of self-harm, e.g., EPDS item 10 > 0) during pregnancy or the postpartum period are eligible to participate in the study. Of note, cutoffs used in deeming a total EPDS score “elevated” vary by site in accordance with site clinical practices. Data are collected at each site for one year. In addition to patient-level data, we also collect qualitative data from clinic providers and staff across all sites, as described below.

### Procedures

#### Pre-implementation of study.

Prior to launching operations at a site, the research team identifies and fosters partnerships with key personnel, including perinatal social workers, nurse and social work managers, departmental leadership, and data management experts. A lead perinatal SW at each site meets with the research team biweekly to review procedures and ensure the comprehensive identification of eligible patients. Within their clinics, lead perinatal SWs also support awareness of the study among patients, providers, and clinic staff. Through conversations with site personnel, study staff also capture details about site-specific screening and referral-related practices (e.g., frequency and format of EPDS administration, staff responsible for flagging elevated scores, how referrals to SW are made and documented), which inform the development and customization of EPIC reporting tools that the study staff utilizes to identify eligible patients.

#### Implementation of study at each site.

Once a site is active, potentially eligible patients are identified for outreach using site-customized EHR reports. These reports return lists of adult patients who have not opted out of research-related contact, have a recent elevated EPDS score, have had a recent routine prenatal or postpartum visit at the relevant site, and who are pregnant or have recently delivered. Study staff conduct detailed reviews of all medical charts to determine eligibility and code relevant clinical encounters according to patient referral status (e.g., referred to SW or not). If patients have an elevated EPDS score but do not receive a referral to SW, the research team codes any information in the EHR pertaining to patient mood or provider rationale for not referring (e.g., discussion of mood, suggestion of SW services, patient declining referral due to existing mental health support). See “EHR” under Data Sources section below for more details.

#### Enrollment of eligible individuals.

MGB policy permits researchers to directly contact potentially eligible patients about research studies unless an individual has specifically opted out of such contact [39]. Eligible individuals are contacted via phone and a preferred contact method (text and email or email only) up to five times to enroll in the study. Contact ceases after any expression of disinterest or non-consent for contact. Upon contact with an interested and eligible individual, the study staff either enrolls the patient via phone call or the patient self-enrolls through a link to an online survey hosted from an encrypted Research Electronic Data Capture (REDCap) [40,41] project. Participants are reimbursed $25 via electronic gift card for each survey they complete. Depending on eligibility, participants may therefore earn a total of $25 (postpartum single-survey arm), $100 (postpartum longitudinal arm), or $125 (pregnancy longitudinal arm) for survey completion.

In the event of endorsement of thoughts of self-harm or suicide (EPDS item 10 > 0), participants are directed to complete the Columbia Suicide Severity Rating Scale (C-SSRS) [42]. If the participant endorses C-SSRS items 3, 4, or 5 (past-week active ideation with specific plan, method, and/or intent), the participant receives a list of relevant resources and notification of upcoming SW contact. An automated alert is immediately sent to the study team and the lead SW at the participant’s site. The SW contacts the participant within two business days to further evaluate the nature and severity of the participant’s suicidal ideation, assess safety, contact local supportive services (ER, law enforcement) as indicated, and revisit resources. The site SW then contacts the participant’s OBGYN provider to ensure care continuation. The encounter is documented in both the patient’s EHR and in the study REDCap database, and the IRB is notified of any suicidal or homicidal ideation (e.g., potential harm to an infant) in accordance with MGB Human Research Committee reporting guidelines.

### Data sources

This study protocol includes qualitative and quantitative data collected from EHR, REDCap surveys, and qualitative interviews (see Table 1 below of data sources mapped to study outcomes).

**Table 1:** RE-AIM evaluation of STEPS for PPD.

RE-AIM dimension	Measures	Data sources	Data type	Level
Reach • The proportion of individuals who receive screening and referral (after elevated EPDS)	• % of individuals screened with EPDS at least 1 time in pregnancy and 1 time in the postpartum • % of individuals with EPDS ≥ 10 * or endorsement (>0) of self-harm item (item 10) who received referral to SW • % of referred patients who attend consultation with SW	EPIC report EPIC report SW referral log	Quantitative	Patient
Effectiveness • Treatment and clinical outcomes following EPDS screening and referral to SW	• % of individuals who report at follow-up timepoints having received treatment (medication, therapy) post-referral • Description of treatment received • EPDS score at 6 months postpartum	REDCap survey	Quantitative Qualitative Quantitative	Patient
Implementation • How screening and referral is implemented across OB sites within the MGB healthcare system	• Description of procedures of screening, referral, and SW procedures at each site • Description of what is working well/could be improved with implementation	Provider interviews	Qualitative	Providers/Clinic
Influencing factors • Contextual factors, barriers, and facilitators influencing Reach, Effectiveness, & Implementation	• CFIR assessment of contextual factors impacting implementation at each site • Barriers and facilitators to reach, effectiveness, and implementation	Provider interviews Provider interviews, patient interviews & survey	Qualitative	Providers/ Patients

*EPDS cut-off for referral to social worker varies by clinic.

#### Edinburgh postnatal depression scale.

The Edinburgh Postnatal Depression Scale (EPDS) is a widely used screening tool designed to identify individuals who may be experiencing depression in pregnancy or the postpartum [19]. The EPDS consists of ten statements related to feelings and mood over the past week, each rated on a four-point scale (from 0 to 3). Total scores can range from 0 to 30, with higher scores indicating a greater likelihood of depression. The following severity ranges have been established for the EPDS [43]: none or minimal depression (0–6), mild depression (7–13), moderate depression (14–19), and severe depression (19–30). Different cut-off scores are recommended based on specific clinic objectives for identifying patients (e.g., identifying more highly symptomatic individuals or as many at-risk individuals as possible). An EPDS cut-off value of ≥ 11 maximizes combined sensitivity and specificity, whereas a cut-off value of ≥ 13 is less sensitive but more specific (i.e., for a depressive episode) [18]. This study captures EPDS scores for eligible patients via EHR and/or a survey, as described below.

#### Electronic health records (EHR).

From EHR reports and chart review, we collect demographic and contact information, available EPDS score, details of any clinical encounter(s), and key psychiatric and obstetrical data. The EHR reports yield preliminary data for all eligible individuals, including those who do not go on to enroll in the study, enabling richer evaluation of patient populations as well as the representativeness (e.g., sociodemographic characteristics and symptom severity) of the subset of patients who enroll. Specific psychiatric and obstetrical data gleaned from EHR review include obstetric history (i.e., lifetime pregnancies, deliveries), weeks pregnant or postpartum at time of the relevant appointment, and any psychiatric diagnoses and active psychotropic medication prescriptions. EHR review also permits assessment of basic eligibility criteria (e.g., fluent in English, not already outreached by study team, currently pregnant or had recent live birth, etc.).

Study staff use EHR data to characterize clinical encounters dichotomously according to referral status (i.e., referred to SW, not referred to SW) and, in cases of non-referral, record relevant appointment features. Example categories include “no evidence of EPDS screening,” “no documentation of elevated score and no discussion of mood,” “documentation of elevated score, but no discussion of mood/score,” “discussion of mood/score, but no evidence SW consult is suggested,” and “evidence that provider suggests SW consult, but no referral placed.” When a referral is not placed despite patient-provider discussion of depressive symptoms, several additional follow-up codes are utilized as needed: “patient reported they are connected with a psychotherapist/mental healthcare provider,” “patient reported they are connected with prescriber or are taking psychiatric medications,” “patient endorsed strong support from family/friends,” “patient felt they were coping well,” “patient denied depressive symptoms or declined referral offered,” etc. Thus, EHR data are used 1) to identify eligible patients with an elevated EPDS score, 2) to categorize eligible patients as either referred or not referred to SW, and 3) to code any relevant characteristics of clinical encounters immediately proximal to a patient’s completion of the EPDS.

#### Patient surveys.

Participant quantitative and qualitative data are collected through patient-level surveys distributed from and stored within a REDCap database. These surveys collect patient-reported data pertaining to topics such as healthcare access (insurance status, travel time to provider, general barriers and facilitators to treatment); social supports (sources and degree of support, maternity leave); pregnancy and infant health; general health and related behaviors (substance use, breastfeeding); and medical, obstetrical and psychiatric history. At each follow-up timepoint in the longitudinal arms of the study, participants are also invited to complete the EPDS to assess for change in depressive symptom severity, report any treatment (e.g., medications, therapy) received for mental health, and describe any barriers and facilitators to accessing treatment. Participants “check all that apply” from a list of barriers (e.g., time, cost) and facilitators (e.g., family/friends, provider support) informed by relevant literature [25,26] and the expertise of the research team. Participants can also select “other” and elaborate in writing upon additional barriers or facilitators to accessing treatment.

#### Social worker referral log.

The lead social worker at each clinic maintains a log of patients referred to them after an elevated EPDS score. The social worker also records all successful or attempted outreaches (e.g., phone, email) and any available patient outcome information (e.g., referred for therapy).

#### Site procedures log.

Upon expansion to a site, the research study team documents procedures pertaining to PND screening and referral, including screening format (e.g., written or digital), EPDS cutoff used, party (e.g., nurse, obstetrician, medical assistant, administrator) responsible for EPDS score flagging and/or review, how (e.g., Epic order or warm hand-off) a referral is made to SW, and what a SW typically does upon receipt of a referral. The log is used to capture similarities and differences in practices and procedures across sites.

#### Semi-structured qualitative interviews.

The Consolidated Framework for Implementation Research (CFIR) is a framework commonly used to direct the assessment of multi-level barriers and facilitators to intervention implementation [34,44]. A systematic review by Hu et al. (2023) [26] established the need for more high-quality studies focused on the CFIR characteristics of available interventions and for future research to be informed by implementation considerations. We therefore plan to use the CFIR framework to inform the development of semi-structured interview guides for use with providers and staff within participating clinics. These interviews will build from previous research on barriers and facilitators [26] by exploring contextual considerations impacting implementation of the intervention package at each site. As all interventions are implemented within a given context, they are necessarily influenced by surrounding social, cultural, political, environmental, and economic factors [45]. Therefore, we will assess all CFIR domains, including intervention characteristics (e.g., “How would you describe screening and referral processes in your clinic?”), outer setting (e.g., “What kinds of local policies or incentives support the depression screening and referral practices at your clinic?”), inner setting (e.g., “How well do procedures for perinatal depression screening and referral fit into the systems of this clinic?”), individual (e.g., “What factors impact patients’ ability to have depression symptoms addressed in OB clinics?”), and process (e.g., “How consistently are procedures for screening with the EDPS and referring to the social worker implemented as intended?”). Invited providers may include any staff involved in the screening and referral intervention cascade (e.g., OB providers, nurses, social workers). We will use snowball sampling to identify key personnel within each site, starting with the site’s lead perinatal social worker. We will utilize purposeful sampling [46] to also identify and interview participants categorized as either having “returned to wellness” or experiencing “sustained depression” (see “effectiveness”; n = 10 for each group) at each of the four sites. Target number of interviews for both patients and providers will be informed by guidelines on saturation for qualitative research [47]. Both provider and patient participants will be compensated with a $75 electronic gift card for interview completion.

## Analytic approach

### RE-AIM framework

Our study is guided by RE-AIM, an implementation and intervention/services evaluation framework that has been applied to a wide range of settings, populations, and health issues [33,48]. RE-AIM dimensions of reach, effectiveness, adoption, implementation, and maintenance can be assessed at both the individual (patient) and multiple additional ecological levels (e.g., staff, provider, clinic, and health systems levels). Our investigation focuses on screening and referral with respect to reach and effectiveness (measured at the patient level) and implementation (measured at the provider and clinic levels) as well as contextual factors, barriers, and facilitators that affect reach, effectiveness, and implementation. We will use an explanatory sequential mixed methods design, described further below, to integrate qualitative and quantitative data. We have collected and plan to analyze quantitative data pertaining to reach and effectiveness (see Table 1) as well as patient-reported barriers to accessing treatment. These data will inform our CFIR-guided follow-up qualitative interview data collection and analysis exploring factors impacting intervention reach, effectiveness, and implementation [38].

#### Reach.

To assess the “reach” of the screening and referral intervention, we will report on the proportion of patients who received screening with the EPDS at least once in pregnancy or at least once in the postpartum during the relevant study timeframe. We will also quantify rates of elevated EPDS scores, indicative of possible PND. Among patients with elevated EPDS scores, we will report on rates of referral to the embedded perinatal social worker as well as rates of patient-SW connection (defined as one or more phone conversations or visits). Rates of referral to and connection with the social worker are captured in the EHR as well as social worker referral logs and will be reported using descriptive statistics.

#### Effectiveness.

To explore the “effectiveness” of the screening and referral intervention, we will report on the proportion of enrolled individuals (i.e., survey completers) who report receiving treatment for depression following an elevated EPDS screen. Treatment data will be extracted from REDCap surveys and include treatment type (therapy, antidepressants, support groups, etc.) and medication details (name, dose, frequency) both prior to pregnancy and after the elevated EPDS screen. Given that any mental health services received outside of the MGB system would be absent from a patient’s EHR, we elected to capture treatment data from self-report rather than chart review.

We will assess symptom improvement, another facet of effectiveness, via scores on a follow-up EPDS submitted by participants 6 months after their routine OB postpartum visit (or, for participants pregnant at the time of their enrollment, 6 months postpartum). We will group participants into either a “return to wellness” group (EPDS ≤ 6, as determined by EPDS cut-off for no or minimal depression) [43] or a “sustained depressive symptoms” group (EPDS ≥ 12, a commonly used threshold for “possible depression”) [49]. We will report on treatment rates and average EPDS scores by cohort and at each timepoint using descriptive statistics, and conduct exploratory predictive analyses to assess factors (e.g., treatment received, demographics) that may predict depressive symptom status (e.g., “return to wellness” versus “sustained depression”) at 6-months postpartum.

#### Implementation.

Through previous pilot work [50] and the pre-implementation phase of this study, we learned that systems for screening and referral vary across MGB system OB clinics. Therefore, we will describe key components of procedures for PND screening and associated referrals to internal social workers, capturing unique variations between clinics (see Table 2).

**Table 2:** Example perinatal depression screening and referral intervention components at Site A.

Clinic	Screening timepoints	Screening measure	How screening administered	Threshold score used to Trigger SW Referral	Monitoring of elevated screening scores	Referral process to SW
**Site A**	1^st^ prenatal appointment and again at 24-28 weeks prenatal. 2 weeks postpartum phone call made by RN 6-week postpartum wellness check 6 months postpartum	EPDS	Electronically via Patient Gateway prior to OB visit OR provided iPad at visit to complete	EPDS ≥ 10 or > 0 on item 10	In-basket automated alert; provider (OB or nurse) reviews EPDS before or during visit or calls patients if no visit scheduled or routes to another provider	EPIC order to SW, In-basket message, or warm handoff

Similarly, we document the clinic-to-clinic variation in procedures and SW interventions provided following referral (see Table 3).

**Table 3:** Example outreach and procedures of perinatal social work by components at Site A.

Clinic	Outreach upon receiving referral	Assessments Conducted	Services Provided	Follow-up
**Site A**	Patient Gateway message, phone calls – outreach until contact made	As determined by clinical judgment: assess mental health history (psychotherapy, medication, previous psychiatric admission); assess SI/HI, patient goals	Depending on patient needs: care navigation; psychoeducation; therapy as needed; referrals to community resources, provider for therapy and/or medication management, support groups	Outreach 3 weeks following contact with patient

### Influencing factors

As described in the “Semi-Structured Qualitative Interviews” section above, we will collect qualitative data to capture contextual factors influencing intervention reach, effectiveness, and implementation among providers and patients. We will invite providers (n = 10 at each site) to participate in semi-structured key-informant interviews guided by CFIR domains and constructs. Patient participants, stratified by remission status, will have the opportunity to complete similar semi-structured interviews guided by patient-level, CFIR-informed questions. We will also analyze participant-reported data from REDCap surveys (see “Patient Surveys”) on barriers and facilitators to care regardless of participant remission status or semi-structured interview participation. In keeping with an explanatory sequential mixed methods design [37,38] preliminary findings on reach, effectiveness, and implementation at a given site will inform the questions asked.

### Data analysis and integration

As described above, findings on reach and effectiveness will be reported using descriptive statistics. We will use rapid qualitative analysis [51] to analyze open text questions about perceived barriers and facilitators captured in the patient REDCap surveys. Advantages of rapid qualitative analysis include its ability to generate findings in a brief time frame relative to other qualitative approaches, thereby facilitating and informing targeted downstream data collection in longitudinal studies with multiple data collection and analysis phases [51–53]. We will use the Planning for and Assessing Rigor in Rapid Qualitative Analysis (PARRQA) framework [54] to guide our analysis process and reporting of findings. In-depth qualitative interviews will be analyzed using the framework method approach, which has been identified as a systematic and flexible approach to management and analysis of qualitative data in health research [55] and consists of several stages, including transcription, familiarization with the data, development of a data coding system, and linking codes to generate overarching categories/themes [56]. A key distinguishing element of the framework method is the use of a matrix consisting of rows (study participants), columns (codes), and cells of summarized data. The matrix allows researchers to develop a coding system iteratively, reducing the data by case and code, and facilitates comparisons and pattern identification [57].

In mixed methods research, integration of qualitative and quantitative data can be implemented at multiple levels, including the study design, methods, and interpretation and reporting levels of research [57]. At the study design level, we primarily utilize an explanatory sequential design [37,38], where we first collect and analyze quantitative data, then use these findings to inform subsequent qualitative data collection and analysis. For example, participant-reported quantitative data (i.e., depressive symptoms at six months postpartum) and quantitative reach and effectiveness data gleaned from EHR will be used to refine the interview guides and inform purposeful sampling for patient interviews. Integration of data will also occur at the interpretation and reporting level [38]; we will report on rates of screening and referral across clinics and, through narrative and joint display approaches, highlight clinic-specific barriers and facilitators to procedure implementation. We will also report on barriers and facilitators to accessing treatment and improvement of depressive symptoms as experienced by participants who have “returned to wellness” or who have “sustained depression” at 6 months postpartum.

## Conclusions

While Perinatal Access Programs with the important goal of improving patient well-being are gaining traction in the United States, data on the effectiveness of PND screening and the outcomes of referrals made for treatment of perinatal psychiatric distress are still limited. This research study aims to provide detailed descriptions of screening and referral procedures as well as treatment outcomes across multiple obstetrics clinics within an integrated academic medical system. We seek to learn how individuals who have elevated depressive symptoms in pregnancy and postpartum are doing over the first year postpartum, how their depressive symptoms change, and what types of treatments or supports they receive. Documenting the variability in screening and referral procedures across clinics facilitates exploration of processes associated with better clinical and implementation outcomes. Our integration of qualitative and quantitative data also facilitates the identification of aspects of the screening and referral cascade which may benefit from modification in a given setting. By using a mixed methods approach in this study, we intend to enrich the understanding of factors contributing to favorable outcomes in perinatal depression screening procedures, referral procedures, and clinical and treatment outcomes.

There are several anticipated challenges and limitations associated with the study approach, population, and setting. First, this is an observational study; hence, the different study groups are not assigned at random. There may be between-group differences in patients and providers that could bias the results. We characterize the clinic populations in detail and consider any differences in conducting our analyses and interpretation of results. The primary goal of this study is to describe in detail different approaches, processes, and outcomes associated with PND screening and referral procedures as they are currently enacted within and across a large health care system. We are thus not seeking to make causal claims of differences in outcomes across clinics, but rather explore differences in a hypothesis-generating manner. Additionally, to minimize respondent burden, we capture as much information as possible through EHR review; however, there are limitations to this approach (e.g., between-provider or between-clinic variation in documentation practices, missing data for treatments received outside of our healthcare system).

Several features of the study may limit its generalizability. This study explores a single, large academic medical system in New England, and findings may not generalize to other health systems with respect to processes, providers, and populations served. Furthermore, engagement of perinatal individuals with elevated depressive symptoms in research can be difficult due to the many demands they are facing (e.g., caring for newborn and other children, limited sleep, increased stress). Hence, there may be biases in who is able and willing to complete surveys or interviews. Additionally, as we utilize EHR data to assess the “reach” domain of CFIR yet rely on a limited sub-set of those patients who enroll and complete surveys to gain insight into clinical outcomes (“effectiveness”), the generalizability of the “effectiveness” data may be limited by their derivation from a significantly smaller sample. However, preliminary insights on the effectiveness domain will inform future research that may utilize alternative data collection methods to observe a greater proportion of patients and more comprehensively understand patients’ long-term mental health outcomes. That the study is conducted in English, that participants must have access to a computer or smart phone at multiple timepoints to complete the surveys, and that there are no specific procedures in place to ensure survey completion represent additional limitations. Finally, factors such as staff turnover or shifting clinical procedures may affect the context of the study over its course. Each of these limitations will be carefully considered in the interpretation and reporting of study findings.

Despite these limitations, the STEPS study is well-positioned to provide meaningful, real-world information on the heterogeneity and complexities of, and outcomes associated with, the implementation of perinatal depression screening and referral programs in large healthcare systems. There is a robust and well-established research literature on validated perinatal depression screening tools and effective treatments, yet there is a substantial gap between this evidence base and systematic implementation in healthcare settings [58]. Findings from this study will characterize, in detail, both promising approaches to perinatal screening and referral service implementation as well as potential shortfalls and challenges of such initiatives, informing best practices and the development of future randomized comparative effectiveness and implementation research studies across diverse obstetrics clinics. Our assessment of contextual factors and barriers to implementation, as guided by CFIR, can then inform the selection of targeted strategies for improvement [58,59]. Future rigorous research designs may include hybrid effectiveness-implementation designs, which blend clinical effectiveness and implementation research to allow for more rapid translational gains, identification of effective implementation strategies and useful information for decision-makers, and examination of statistical differences in clinical outcomes across sites [60]. In short, STEPs for PPD will structure an internal and external “scan” of PND screening-to-referral procedures and outcomes in MGB obstetrics clinics. Such a scan is an important element in the development of a Learning Health System [61,62], in which data are used to improve patient care through the continuous learning and improvements made possible through bidirectional research-practice feedback loops. We ultimately envision that the STEPs for PPD project and findings could inform the development of a Learning Health System for perinatal mental health implemented across MGB and serve as a model that could be disseminated to other health systems nationwide.
